# Analysis of oligomers to assess exposure to microplastics from foods. A perspective

**DOI:** 10.3389/fnut.2023.1186951

**Published:** 2023-05-22

**Authors:** Emmanouil D. Tsochatzis, Georgios Theodoridis, Milena Corredig

**Affiliations:** ^1^Department of Food Science, CiFOOD, Centre for Innovative Foods, Aarhus, Denmark; ^2^Center for Interdisciplinary Research and Innovation (CIRI-AUTH), Thessaloniki, Greece; ^3^Department of Chemistry, Aristotle University of Thessaloniki, Thessaloniki, Greece

**Keywords:** microplastics, nanoplastics, oligomers, foods, exposure, risks, safety

## Abstract

There is an emerging interest in evaluating the presence of microplastic (MP) and nanoplastic (NP) residues in food. Despite their potential threat to human health, there is still a need for harmonized methods to evaluate and quantify their presence. Incomplete polymerization may occur during the production of plastic. Conversely, oligomers are formed during chemical, mechanical, or enzymatic depolymerization. Oligomers are a few nanometers in size. Recent advances in analytical chemistry have enabled the quantification and identification of these oligomers in various complex biological matrices. Therefore, we propose that the specific nanosized oligomers can be considered markers for the presence of MPs/NPs. This advance may facilitate a broader perspective for the assessment of MPs/NPs exposure, leading to the evaluation of food safety and associated risks to humans.

## 1. Background: microplastics and nanoplastics in food

The production of plastics for food packaging continues to increase, with more than 20 billion kilos of total plastic demand in Europe used for packaging (1). According to a recent report, the most demanded plastic polymers in the food industry in Europe in 2018 were polypropylene (PP, 19%), high-density polyethylene (HDPE, 12%), low-density polyethylene (LDPE, 18%), and polyethylene terephthalate (PET, 6%) (2). Polyethylene terephthalate (PET) is one of the most important and widely used polymers as a food contact material, either virgin or recycled (3).

The small fragments derived from the degradation of plastic material, defined as microplastics (MP) or nanoplastics (NP), have a hidden impact on the environment, human health, and ecosystems (4, 5). The term “*MPs*” is loosely employed to refer to all of these plastic particles. However, the size of MPs is usually between 1 μm and 5 mm (6, 7), whereas NPs are particles in the nanometer range, between 1–500 nm (7). The presence of NPs in food or food contact materials may be intended, for example, to create biosensing devices or engineered functional particles.

Laboratory simulations have shown that plastic MPs and NPs can be generated by the fragmentation and degradation of plastic material (8) and can be released from products containing them as functional components, such as textiles or cosmetics (9). Plastic-derived material at the nano-and microscale has been identified in human lung tissue (10), placenta (11), and blood (12), although without a clear description of the pathways involved (13).

Assessing the risk of MPs and NPs to our health *via* the food chain requires a thorough understanding of how much of these micro-or nano-sized structures are leached into the environment, when and how they enter the food chain, where they partition, and what their fate is. Analytical methods such as spectroscopy, molecular imaging, hyphenated techniques combined with mass spectrometry such as time-of-flight mass spectrometry (TOF-MS), pyrolysis with GC–MS, light scattering, or microscopic techniques are being evaluated to selectively detect MPs and NPs in our food products (14). In food, the complexity of the matrix poses a substantial challenge to their detection and quantification.

**Figure 1:**
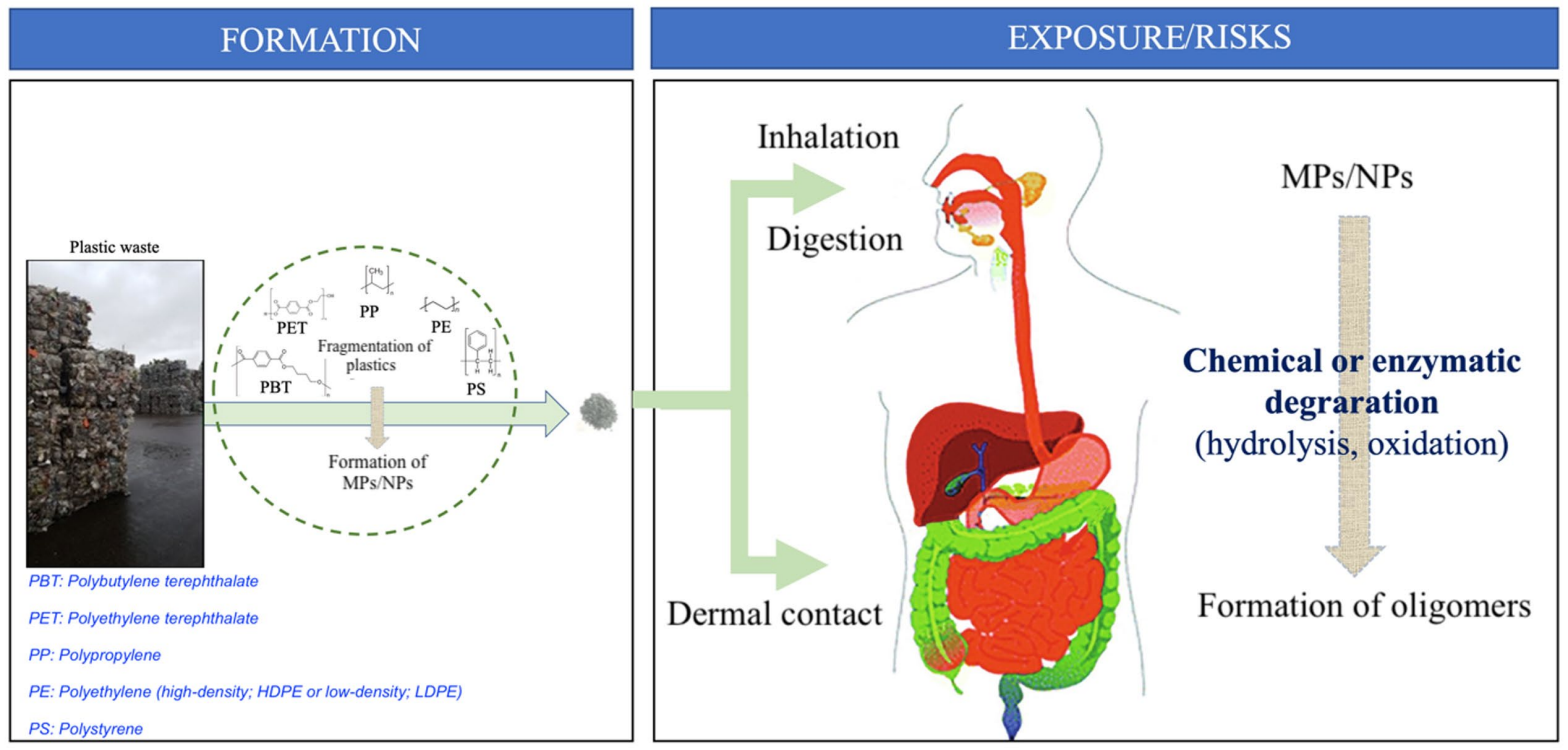
MP/NP formation leads to human exposure and associated risks. Consumption (ingestion, inhalation, or dermal contact) of MPS/NPs would lead to an *in vivo* chemical or enzymatic degradation leading to the formation of corresponding oligomers.

When present in food, MPs and NPs are considered to be products of plastic degradation or cross-contamination, which can leach and be ingested and processed in the upper gastrointestinal tract (Figure 1) and further fermented by the gut microbiota (15). It has been recently demonstrated that insects can metabolize and degrade plastics through the activity of their gut microbiota (16, 17). The digestive tract has a very high surface area due to its absorptive function and provides a very efficient route for orally ingested particles to enter the body. In pharmaceutical science, many oral delivery formulations are based on polymeric nanoparticles (18), as they pass through the intestinal mucosa quickly and protect the bioactive materials from degradation by enzymes and acids during transit. Studies on the design of nanoparticles for oral delivery provide a wealth of knowledge on how to improve transport across the gastrointestinal and blood–brain barriers with a low risk of inflammation (19). The size of these particles affects their absorption, and sizes larger than 10 mm do not appear to readily penetrate the mucus layer (18).

The gastrointestinal epithelium is designed to protect us from foreign substances. These cells are the last barrier to absorption; they are cylindrical in shape and connected to each other through tight junctions, with a brush border membrane on top, covered by a dense layer of mucus. After digestion, MPs or NPs must first diffuse through the mucus layer and then enter the bloodstream. In a recent study, fluorescent-labeled model nanoparticles were used to investigate the effect of basic physical parameters such as size, charge, and hydrophobicity on the ability to cross the intestinal barrier (20). The physical properties of the polymeric material have an impact on bioadhesion, interactions with the mucus layer, and cell absorption. Factors such as size, negative charge, and hydrophilicity have been shown to facilitate the passage of nano- and microplastic particles through the mucus layer. Once through the mucus layer, surface charge and hydrophobicity are shown to improve the process of trans-intestinal transport (20). Trans-cellular transport of the plastic NPs is mostly dependent on endocytosis, whereby the particles are transported through the cell and released at the basolateral membrane (21–23). Particles smaller than 4 mm interact with the intestinal layer (20).

The pathophysiological consequences of acute and chronic exposure to MPs, NPs, and oligomers, in the mammalian system, particularly in humans, are still unclear. Disruptive effects of MPs and NPs have been extensively documented in marine invertebrates and mice (24), but not in humans. It has been shown that MPs and NPs cause an imbalance in the gut microbiome flora, with a reduction of beneficial bacteria and an increase in harmful bacteria (25). The latter could affect the intestinal barrier, and certain bacterial products or species could enter the bloodstream and cause damage to other tissues and organs (25). In addition, the continued presence of low levels of MPs and NPs in foods may be increasingly harmful to individuals with an irritable bowel disorder due to their increased intestinal permeability.

## 2. Plastic oligomers are nano-sized

Low molecular weight oligomers (<1,000 Da), formed as by-products of incomplete polymerization during manufacturing, can derive from the presence of impurities in the raw materials used for the production of plastic materials. At the moment, no official definition for oligomers exists, nor is there any way to distinguish them from the physicochemical behavior of polymers. Furthermore, they can also result from the degradation and depolymerization of high molecular weight polymers, whether enzymatically or chemically (3, 26–28).

**Figure 2:**
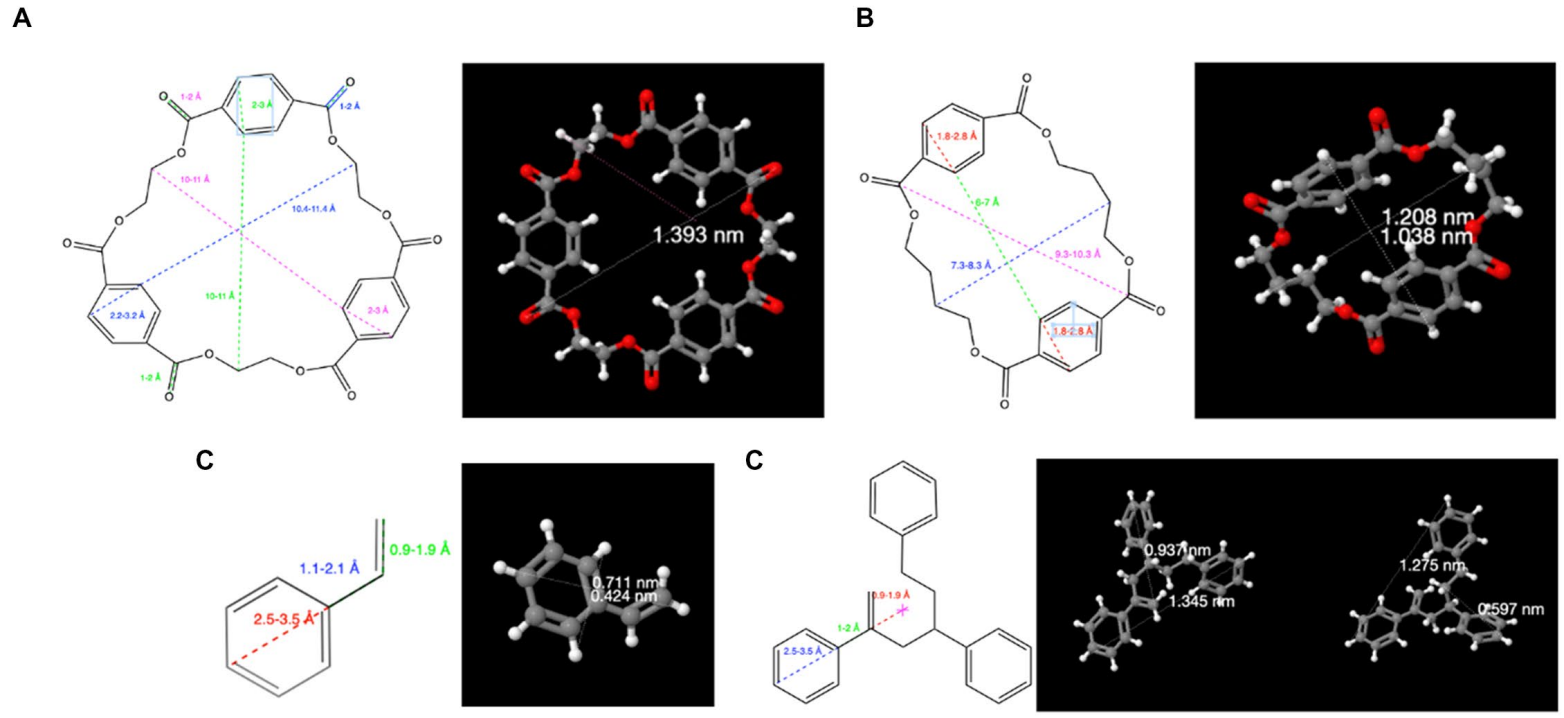
Calculated dimensions with selected IT tools for **(A)** PET cyclic trimer; **(B)** PBT cyclic dimer; **(C)** styrene monomer; **(D)** styrene trimer (2,4,6-triphenyl-1-hexene). Oligomers of PET, PBT, and PS can be considered NPs because they have molecular dimensions larger than 1 nm.

*In silico* calculations demonstrate that oligomers have nanosized dimensions. For example, using available software (ChemDraw, Perkin Elmer) and the online IT database Chemspider,^1^ it can be easily concluded that PET and PBT cyclic polyester oligomers, in addition to PS oligomers, have sizes larger than 1 nm. PET 1^st^ cyclic oligomers have dimensions ranging from 0.9 nm for the dimer (*M* = 384.1 Da) to 1.6 nm for the heptamer (*M* = 1344.3 Da) (Figure 2A). Similarly, the PBT cyclic oligomers range from 1.0 nm (dimer; *M* = 440.4 Da) up to 1.9 nm (pentamer; *M* = 1101.1 Da) (Figure 2B).

The monomers styrene (Figure 2C) and acetophenone (the styrene oxidation product) are smaller molecules, but their oligomers are larger than 1 nm, as shown in Figure 2D for the styrene trimer. In addition, as recently reported (29), the size of PS oligomers may vary due to the formation of molecules of different sizes, such as acetophenone, 1,2-diphenylcyclobutane, 1a-tetralin, 2,4-diphenyl-1-butene, 1,1-diphenylethylene, and 2,4,6-triphenyl-1-hexene.

Oligomers have been detected in plastics, food, and human blood (12, 26, 27, 30) using high-resolution mass spectrometry (HR-MS). These works are a step toward the development of quantification methods for complex matrices. However, challenges still exist for their identification and structural elucidation due to the lack of commercially available analytical standards (3).

Oligomers have been shown to migrate from virgin or recycled plastic packaging materials (3, 31) to food. Similarly, oligomers have been shown to leach onto the surface of fibers during the dyeing process (32). In the case of food and its packaging, recycling technologies may have an impact on the generation of oligomers or other NPs and their subsequent migration and safety, but no details are available in this regard. Furthermore, the presence of these oligomers or the formation of NPs in new biobased plastic materials is also unknown.

No information is available on the toxicity of oligomers except for limited *in silico* assessments for PET and PS (29, 33). In the case of polyesters, cyclic oligomers have only been assessed based on QSAR models (27). PET and PBT cyclic oligomers are already classified as Cramer III toxicity classes, while the corresponding linear oligomers are of low toxicity (Cramer I) (26, 27). As mentioned above, the limited availability of analytical standards limits full detection and quantification (28). In the case of PS oligomers, a recent IARC report categorizes them as potentially carcinogenic, together with their monomer (styrene) (34). All these considerations must certainly be reflected in the respective Tolerable Daily intake (TDI), based on the Toxicological Threshold of Concern (TTC) (35).

Oligomers are the smallest components of the NP family and can be readily transferred to the blood. A recent modeling study supported passive diffusion as the mechanism of cellular transfer for plastic oligomers (36). However, apart from *in silico* toxicological assessment, no *in vivo* or *in vitro* data exist on the potential effects of exposure to either NPs or oligomers, as they can also be considered NPs. The study of potential human health effects is at an early stage of development, and the assessment of human exposure is considered to be very challenging. What are the main routes of exposure? The levels used in acute experiments are often unrealistic concentrations in the case of food. However, the effects of long-term exposure through food are largely unknown.

## 3. Conclusion

There is increasing evidence that humans are exposed to MPs and NPs; however, a more quantitative understanding is needed to assess the major sources of exposure. The presence of trace plastic oligomers in the blood raises questions regarding their potential origin, whether degraded plastic, MPs, or NPs. In this respect, more dedicated and multidisciplinary research is needed to verify the respective biochemical pathways involved.

Assessing the risks posed by oligomers as NPs will depend on addressing important questions, namely, how to calculate/measure oligomers/NPs released from plastics in food or the environment, identify their sources, evaluate how they may interact with themselves or with other components at the molecular or colloidal level, demonstrate their fate through ingestion, and identify their impact on human health. For this, it is essential to establish sensitive and selective analytical techniques to identify and quantify them.

Oligomers, as the smallest members of the NP family, should be considered markers of NP exposure. They are suitable as model compounds representing the presence of NPs in the respective materials. Specific target oligomers can facilitate the quantification and assessment of MPs/NPs. They can be considered excellent markers to evaluate the presence of plastic depolymerization in food, the environment, and human biological samples. Recent advances in analytical methods now make it possible to assess their presence and better evaluate the risk of exposure. This approach should be encouraged to allow for progress toward a safer food value chain.

## Data availability statement

The original contributions presented in the study are included in the article, further inquiries can be directed to the corresponding author.

## Author contributions

ET, GT, and MC contributed to the study design. ET structured and wrote the first draft of the manuscript. GT and MC wrote sections of the manuscript. All authors contributed to the article and approved the submitted version.

## Conflict of interest

The authors declare that the research was conducted in the absence of any commercial or financial relationships that could be construed as a potential conflict of interest.

## Publisher’s note

All claims expressed in this article are solely those of the authors and do not necessarily represent those of their affiliated organizations, or those of the publisher, the editors and the reviewers. Any product that may be evaluated in this article, or claim that may be made by its manufacturer, is not guaranteed or endorsed by the publisher.

## Author disclaimer

The manuscript does not represent, and is not intended to represent, an EFSA (European Food Safety Authority) opinion.

## Footnotes

^1^ www.chemspider.com

## References

[1] ING Economics Department. Plastic packaging in the food sector -six ways to tackle the plastic puzzle. (2019) Available at: https://assets.ing.com/m/5d2942da1fbe6ec9/original/ING_EBZ_plastic-packaging-in-the-food-sector.pdf (Accessed April 10, 2023)

[2] Plastics Europe. Plastics –The facts 2020. An analysis of European plastics production, demand and waste data. (2020) Available at: https://plasticseurope.org/wp-content/uploads/2021/09/Plastics_the_facts-WEB-2020_versionJun21_final.pdf (Accessed March 3, 2022)

[3] Tsochatzis ED Lopes JA Corredig M. Chemical testing of mechanically recycled polyethylene terephthalate for food packaging in the European Union. Resour Conserv Recycl. (2022) 179:106096. doi: 10.1016/j.resconrec.2021.106096

[4] Ivleva NP. Chemical analysis of microplastics and Nanoplastics: challenges, advanced methods, and perspectives. Chem Rev. (2021) 121:11886–936. doi: 10.1021/acs.chemrev.1c00178, PMID: 34436873

[5] Ding Y Zhang R Li B Du Y Li J Tong X. Tissue distribution of polystyrene nanoplastics in mice and their entry, transport, and cytotoxicity to GES-1 cells. Environ Pollut. (2021) 280:116974. doi: 10.1016/j.envpol.2021.116974, PMID: 33784569

[6] Belz S Bianchi I Cella C Emteborg H Sirio Fumagalli F Geiss O. Current Status of the Quantification of Microplastics in Water. Results of a JRC/BAM inter-Laboratory Comparison Study on PET in Water. [JRC Technical Reports]. Ispra: European Commission: European Commission. Joint Research Centre (2021).

[7] Picó Y Barceló D. Analysis and prevention of microplastics pollution in water: current perspectives and future directions. ACS Omega. (2019) 4:6709–19. doi: 10.1021/acsomega.9b00222, PMID: 31459797 PMC6648735

[8] Gigault J Pedrono B Maxit B Halle AT. Marine plastic litter: the unanalyzed nano-fraction. Environ. Sci. Nano. (2016) 3:346–50. doi: 10.1039/C6EN00008H

[9] Hernandez LM Yousefi N Tufenkji N. Are there Nanoplastics in your personal care products? Environ Sci Technol Lett. (2017) 4:280–5. doi: 10.1021/acs.estlett.7b00187

[10] Jenner LC Rotchell JM Bennett RT Cowen M Tentzeris V Sadofsky LR. Detection of microplastics in human lung tissue using μFTIR spectroscopy. Sci Total Environ. (2022) 831:154907. doi: 10.1016/j.scitotenv.2022.154907, PMID: 35364151

[11] Ragusa A Svelato A Santacroce C Catalano P Notarstefano V Carnevali O. Plasticenta: first evidence of microplastics in human placenta. Environ Int. (2021) 146:106274. doi: 10.1016/j.envint.2020.106274, PMID: 33395930

[12] Diamantidou D Mastrogianni O Tsochatzis E Theodoridis G Raikos N Gika H. Liquid chromatography-mass spectrometry method for the determination of polyethylene terephthalate and polybutylene terephthalate cyclic oligomers in blood samples. Anal Bioanal Chem. (2022) 414:1503–12. doi: 10.1007/s00216-021-03741-6, PMID: 35024915

[13] Leslie HA van Velzen MJM Brandsma SH Vethaak AD Garcia-Vallejo JJ Lamoree MH. Discovery and quantification of plastic particle pollution in human blood. Environ Int. (2022) 163:107199. doi: 10.1016/j.envint.2022.107199, PMID: 35367073

[14] EFSA Panel on Contaminants in the Food Chain (CONTAM). Presence of microplastics and nanoplastics in food, with particular focus on seafood. EFS2. (2016) 14:4501. doi: 10.2903/j.efsa.2016.4501 PMC11847996 40007823

[15] Tamargo A Molinero N Reinosa JJ Alcolea-Rodriguez V Portela R Bañares MA. PET microplastics affect human gut microbiota communities during simulated gastrointestinal digestion, first evidence of plausible polymer biodegradation during human digestion. Sci Rep. (2022) 12:528. doi: 10.1038/s41598-021-04489-w, PMID: 35017590 PMC8752627

[16] Tsochatzis ED Berggreen IE Vidal NP Roman L Gika H Corredig M. Cellular lipids and protein alteration during biodegradation of expanded polystyrene by mealworm larvae under different feeding conditions. Chemosphere. (2022) 300:134420. doi: 10.1016/j.chemosphere.2022.134420, PMID: 35367488

[17] Tsochatzis ED Berggreen IE Nørgaard JV Theodoridis G Dalsgaard TK. Biodegradation of expanded polystyrene by mealworm larvae under different feeding strategies evaluated by metabolic profiling using GC-TOF-MS. Chemosphere. (2021) 281:130840. doi: 10.1016/j.chemosphere.2021.130840, PMID: 34023760

[18] Babadi D. Nanoformulation strategies for improving intestinal permeability of drugs_ a more precise look at permeability assessment methods and pharmacokinetic properties changes. J Control Release. (2020) 41:669–709. doi: 10.1016/j.jconrel.2020.02.041 32112856

[19] Ahlawat J Barroso GG Asil SM Alvarado M Armendariz I Bernal J. Nanocarriers as potential drug delivery candidates for overcoming the blood–brain barrier: challenges and possibilities. ACS Omega. (2020) 5:12583–95. doi: 10.1021/acsomega.0c01592 32548442 PMC7288355

[20] Guo S Liang Y Liu L Yin M Wang A Sun K. Research on the fate of polymeric nanoparticles in the process of the intestinal absorption based on model nanoparticles with various characteristics: size, surface charge and pro-hydrophobics. J Nanobiotechnol. (2021) 19:32. doi: 10.1186/s12951-021-00770-2, PMID: 33499885 PMC7839302

[21] Yin Win K Feng S-S. Effects of particle size and surface coating on cellular uptake of polymeric nanoparticles for oral delivery of anticancer drugs. Biomaterials. (2005) 26:2713–22. doi: 10.1016/j.biomaterials.2004.07.050, PMID: 15585275

[22] Crecente-Campo J Guerra-Varela J Peleteiro M Gutiérrez-Lovera C Fernández-Mariño I Diéguez-Docampo A. The size and composition of polymeric nanocapsules dictate their interaction with macrophages and biodistribution in zebrafish. J Control Release. (2019) 308:98–108. doi: 10.1016/j.jconrel.2019.07.011, PMID: 31306677

[23] Walczak AP Hendriksen PJM Woutersen RA van der Zande M Undas AK Helsdingen R. Bioavailability and biodistribution of differently charged polystyrene nanoparticles upon oral exposure in rats. J Nanopart Res. (2015) 17:231. doi: 10.1007/s11051-015-3029-y, PMID: 26028989 PMC4440892

[24] Jin H Ma T Sha X Liu Z Zhou Y Meng X. Polystyrene microplastics induced male reproductive toxicity in mice. J Hazard Mater. (2021) 401:123430. doi: 10.1016/j.jhazmat.2020.123430, PMID: 32659591

[25] Yin K Wang Y Zhao H Wang D Guo M Mu M. A comparative review of microplastics and nanoplastics: toxicity hazards on digestive, reproductive and nervous system. Sci Total Environ. (2021) 774:145758. doi: 10.1016/j.scitotenv.2021.145758

[26] Alberto Lopes J Tsochatzis ED Karasek L Hoekstra EJ Emons H. Analysis of PBT and PET cyclic oligomers in extracts of coffee capsules and food simulants by a HPLC-UV/FLD method. Food Chem. (2021) 345:128739. doi: 10.1016/j.foodchem.2020.128739, PMID: 33333359 PMC7896039

[27] Tsochatzis ED Alberto Lopes J Kappenstein O Tietz T Hoekstra EJ. Quantification of PET cyclic and linear oligomers in teabags by a validated LC-MS method – in silico toxicity assessment and consumer’s exposure. Food Chem. (2020) 317:126427. doi: 10.1016/j.foodchem.2020.126427, PMID: 32092611

[28] Tsochatzis ED Lopes JA Holland MV Reniero F Emons H Guillou C. Isolation, characterization and structural elucidation of Polybutylene terephthalate cyclic oligomers and purity assessment using a 1H qNMR method. Polymers. (2019) 11:464. doi: 10.3390/polym11030464, PMID: 30960448 PMC6473883

[29] Tsochatzis ED Gika H Theodoridis G. Development and validation of a fast gas chromatography mass spectrometry method for the quantification of selected non-intentionally added substances and polystyrene/polyurethane oligomers in liquid food simulants. Anal Chim Acta. (2020) 1130:49–59. doi: 10.1016/j.aca.2020.07.018, PMID: 32892938

[30] Tsochatzis ED Alberto Lopes J Gika H Kastrup Dalsgaard T Theodoridis G. Development and validation of an UHPLC-qTOF-MS method for the quantification of cyclic polyesters oligomers in pasta by applying a modified QuEChERS clean-up. Food Chem. (2021) 347:129040. doi: 10.1016/j.foodchem.2021.129040, PMID: 33484960

[31] Gerassimidou S Lanska P Hahladakis JN Lovat E Vanzetto S Geueke B. Unpacking the complexity of the PET drink bottles value chain: a chemicals perspective. J Hazard Mater. (2022) 430:128410. doi: 10.1016/j.jhazmat.2022.128410, PMID: 35295000

[32] Stark M. Comment on “characterization of Nanoplastics, fibrils, and microplastics released during washing and abrasion of polyester textiles.”. Environ Sci Technol. (2022) 56:10543–4. doi: 10.1021/acs.est.1c08880 35762687

[33] Ubeda S Aznar M Rosenmai AK Vinggaard AM Nerín C. Migration studies and toxicity evaluation of cyclic polyesters oligomers from food packaging adhesives. Food Chem. (2020) 311:125918. doi: 10.1016/j.foodchem.2019.125918, PMID: 31869647

[34] EFSA Panel on Food Contact Materials, Enzymes and Processing Aids (CEP) Silano V Barat Baviera JM Bolognesi C Chesson A Cocconcelli PS. Assessment of the impact of the IARC monograph Vol. 121 on the safety of the substance styrene (FCM no 193) for its use in plastic food contact materials. EFS2. (2020) 18:e06247. doi: 10.2903/j.efsa.2020.6247, PMC7586998 33133270

[35] EFSA Scientific Committee More SJ Bampidis V Benford D Bragard C Halldorsson TI. Guidance on the use of the threshold of toxicological concern approach in food safety assessment. EFS2. (2019) 17:e05708. doi: 10.2903/j.efsa.2019.5708, PMID: 32626331 PMC7009090

[36] Järvenpää J Perkkiö M Laitinen R Lahtela-Kakkonen M. PE and PET oligomers’ interplay with membrane bilayers. Sci Rep. (2022) 12:2234–8. doi: 10.1038/s41598-022-06217-4, PMID: 35140293 PMC8828856

